# Comparative analysis of primary health care attributes between children under and over 3 years of age using the primary care assessment tool

**DOI:** 10.1016/j.clinsp.2024.100353

**Published:** 2024-04-04

**Authors:** Luciana Harumi Miranda Omori, Deoclecio Avigo, Itamar de Souza Santos, Gustavo Diniz Ferreira Gusso, Maria Teresa Bechere Fernandes

**Affiliations:** Faculdade de Medicina da Universidade de São Paulo (FMUSP), São Paulo, SP, Brazil

**Keywords:** Primary health care, Pca tool, Child Health

## Abstract

**Introduction:**

Child health actions in Brazil have their primary focus on early childhood. A new epidemiological profile is emerging for children after the first one thousand days: an increase in non-communicable chronic diseases. This research aimed to analyze the attributes of Primary Health Care comparatively among different age groups, using three years of age as the cutoff point.

**Methods:**

The study design was cross-sectional and conducted in three Primary Health Care Units and three Ambulatory Medical Assistance facilities in the Western Region of São Paulo. The PCA Tool Brazil was used as the assessment instrument.

**Results:**

A total of 311 interviews were conducted with caregivers of children aged 0 to 12 years; 153 children were under three years old, and 158 were three years or older. The attributes that showed statistically significant differences between age groups (< 3 years and > 3 years) were affiliation (4.9 × 3.8), longitudinality (5.7 × 5.2), information system (7.4 × 6.3), and services provided (5.4 × 4.5). Through linear regression analysis, it was observed that there was a trend for better overall and essential scores in the evaluations of the group of children under three years old who attended Primary Health Care Units.

**Conclusion:**

The comparative analysis of Primary Health Care attributes among pediatric age groups revealed a trend towards higher scores, according to caregivers' perceptions, for children under three years old. This study suggests the need for the implementation of programs that can better address the healthcare needs of children beyond early childhood.

## Highlights

• Government programs primarily focus on early childhood.
• Primary Care services tend to be better for children under three years old.
• New programs are needed to provide follow-up care for children beyond early childhood.

## Introduction

Child health is a priority area in population care. For their proper development, in addition to knowledge about the characteristics of morbidity and mortality indicators, it is necessary to understand their biological, demographic, and socioeconomic aspects. The set of actions currently in Brazil has a strong emphasis on children in early childhood (especially in the first two years of life), with the National Policy for Comprehensive Child Health Care (NPCCHC) standing out among them. [1,2]

The relevance of early childhood for a child's proper development is well known, and efforts aiming for better health during this period are justified. Despite being a country with heterogeneous realities, the primary indicator reflecting the improvement in the quality of child healthcare resulting from the strengthening of Primary Health Care (PHC) in Brazil is the infant mortality rate. This rate has shown a significant decrease in recent decades, dropping from 29 in 2000 to 12 per 1000 live births in 2021. [3,4]

However, the increase in non-communicable chronic diseases is a reality not only for adults but is also increasingly affecting the pediatric age group, from preschoolers to adolescents. This is due to the increase in obesity and its comorbidities, behavioral problems, and mental health issues (which also impact academic performance). [5,6]

The percentage of Brazilian preschoolers with excess weight (obesity and overweight) is 10%. [5] For Brazilian children and adolescents, there is a prevalence of 12%‒14% for Metabolic Syndrome (METS), [7] 14% for high blood pressure, [8] and 20% for mental health disorders. [6] Strategic Axis IV of the NPCCHC, [1] focuses on Comprehensive Care for Children with Prevalent Childhood Illnesses and Chronic Diseases, and outlines actions for some of these issues. However, in the practice of Primary Health Care Units (PHCUs), these issues often go unnoticed by the healthcare team. Additionally, the families of these children typically have little knowledge about these conditions during this phase of life.

Another point to highlight, as envisioned in the NPCCHC, is the intersectoral actions, especially relevant for promoting health and preventing the previously mentioned morbidities. One noteworthy intersectoral program is the “Health in Schools” program, established in 2007 through Decree 6286 as a federal program. This program aims to create agreements between PHCUs and schools, collaborating on various aspects of primary healthcare. Despite being considered a potent program, it is predominantly used for curative actions rather than preventive actions. [9]

In Brazil, the construction of PHC began in the 1920s, and expanded in 1988 with the promulgation of the Federal Constitution in Brazil and the establishment of the Unified Health System (UHS). It was organized from the 1990s into family health programs that became known as the Family Health Strategy (FHS) from 2006 onwards. The Ministry of Health, in addition to organizing UHS, oversees its operation. In Primary Care, guiding principles are based on the attributes described by Starfield [10] and its assessment can be conducted by Primary Care Assessment Tool-Brazil (PCA Tool). 11, 12, 13

The PCA Tool was developed by Starfield and colleagues, based on Donabedian's model for assessing service quality (structure, process, and outcomes). [14] It filled a gap in the scientific literature since there were few studies evaluating the performance of PHC until then. This instrument is in the public domain and has been adopted by the World Health Organization (WHO). Moreover, it has been adapted and validated in different countries such as Brazil, 15, 16, 17 South Korea, [18] and Spain. [19]

Despite the significant advances related to child health, various studies show that the evaluation of its action in PHC is deficient. [20] There is a shortage of government programs and public health measures for this new epidemiological scenario of child health, which raises concerns in society about the health of children aged three years or older.

Therefore, this study aims to analyze whether there is a difference in the evaluation of PHC attributes among caregivers responsible for children in different age groups, emphasizing the importance of a detailed look from the age of three onwards.

## Materials and methods

The present study had a cross-sectional design and followed the STROBE Statement. It was conducted in PHCUs and Ambulatory Medical Assistance (AMA) facilities in the Western Region of the city of São Paulo, under the responsibility of the Technical Health Supervision of Butantã. The Butantã Regional Municipality is one of the 32 regional municipalities in the city of São Paulo, responsible for an area of 56.1 km^2^ and inhabited by approximately 428 thousand people, with a population density of 7633 inhabitants/km^2^, and a Human Development Index (HDI) of 0.885, the eighth highest in the city. [21]

PHCUs are responsible for a coverage area established by the municipal health technical supervision, with the responsibility of providing longitudinal care to the residents in their territory through the FHS program. On the other hand, AMAs serve users regardless of their address, providing open access for acute demands and serving as a reference for spontaneous demand for a group of PHCUs near their territory.

Data collection took place between 2014 and 2015 in the following Primary Health Care Units: Jardim Boa Vista, Jardim D'Abril, and Vila Dalva, and in the Ambulatory Medical Assistance services: AMA Paulo VI, AMA Vila Nova Jaguaré, and AMA Vila Sônia. This research assessed users of health services, PHCUs, and AMAs using the PCA Tool-Brazil instrument.

The data analyzed in this study belong to the project “Evaluation of Primary Health Care Units, Family Health Teams, and Ambulatory Medical Assistance using the Primary Care Assessment Tool”. The research was submitted and approved by the Ethics and Research Committees of the City of São Paulo and the School of Medicine of USP. CAAE: 32,815,414.3.0000.0065.

### Population and sample

The study targeted the users of the health units mentioned earlier. Interviewers (trained scholarship holders) conducted the research at the entrance of each of these health services, inviting the caregiver accompanying the child who was leaving the service (not necessarily medical care) of the evaluated PHCUs/FHS and AMAs. All children could participate in the study, regardless of their health condition.

This study used data related to children aged 0 to 12 years. A total of 311 interviews were conducted, and 158 (50.8%) of these children were aged three or older.

It states that in the original study, adult participants [22] were included, while in the current study, only data related to children were used. To validate the sample, a “sample power calculation” was performed, considering the total number of evaluated children (311), with a 5% alpha error, a score difference between age groups of 0.15, and a standard deviation of 0.38. [23] Therefore, the statistical power for this sample was determined to be 80%.

### Exclusion criteria

Users who had never been attended to at the health unit where they were being interviewed or who were accompanied by minors under the age of 18 were excluded.

### Instruments

The instrument used was the PCA Tool-Brazil child version, published by the Ministry of Health in 2010. The calculation of the assessment scores followed the PCA Tool Manual. [11]

The socio-demographic data collected included the gender and age of the interviewer and the child, the interviewer's level of education, and economy class, according to the socioeconomic questionnaire of the Brazil Economy Classification Standard/2008. [24] According to the Brazilian system, education is categorized into 5 levels: 1) Incomplete elementary school (less than five years of education), 2) Complete elementary school (five complete years of education), 3) Complete middle school (nine years of education), 4) Complete high school (twelve years of education), and 5) Bachelor's degree.

### Calculation of pca tool scores

The PCA Tool was created by Starfield, Shi, and colleagues [13] as an assessment tool for the structures and processes of PHC, measuring its attributes from the perspective of healthcare professionals and users. [25] It was validated in Brazil in 2006 by Harzheim and colleagues. 15, 16, 17 The child version questionnaire assesses 55 items distributed into 10 components (A‒J) related to the attributes of PHC.

A. Affiliation: identification of the user with the service and identification of a service or professional (doctor/nurse) as the reference for the patient's healthcare.

B. First Contact - Utilization: the extent of access for utilization (appointments, referrals to/from specialists, acute complaints).

C. First Contact - Accessibility: service structure, including location and hours.

D. Longitudinality: temporal continuity of healthcare services, including an interpersonal relationship that expresses mutual trust between users and professionals.

E. Coordination - Care Integration: coordination between different services and actions (referral/counter-referral).

F. Coordination - Information Systems: medical record keeping and access to this information by the caregiver.

G. Completeness - Available Services: services present in the unit or offered by it.

H. Completeness - Services Provided: services received or offered (including preventive and health promotion guidance).

I. Family Orientation: recognition of family factors in determining an individual's health and disease treatment, considering the family as the subject of care.

J. Community Orientation: recognition of environmental and community factors in determining health and disease treatment (including home visits and invitations to participate in Health Councils).

The calculation of scores followed the recommendations of the PCA Tool-Brazil Application Manual [11] and is described in detail in Deoclecio Avigo's Doctoral Thesis – “Evaluation of the attributes of primary healthcare in two coexisting models in the western region of the city of São Paulo, using the Primary Care Assessment Tool”. [22]

### Statistical analysis

A global analysis of pediatric data was conducted according to the child's age group. Categorical variables were presented as counts and proportions. Comparison between groups with ages under and over three years was performed using the Chi-Square test for categorical variables. Quantitative variables were expressed as means and standard deviations, and comparisons between groups were made using analysis of variance (ANOVA).

The subsequent step involved comparing scores between AMAs for children under and over three years of age, as well as between PHCUs/FHS for the same pediatric age groups, using analysis of variance (ANOVA).

Subsequently, a linear regression model was developed to identify a relationship between the overall and essential scores and the type of healthcare unit (AMA or PHCU/FHS), considering the age cutoff of three years for children. Adjustment variables for the model included the age of the caregiver (equal or over thirty years), the sex of the child and the caregiver, as well as the caregiver's education (with the cutoff point being the completion of high school).

For statistical analysis, the R software (R Project for Statistical Computing) [26] was used, with a significance level set at 0.05.

## Results

A total of 311 interviews were conducted, with 153 interviews involving children under the age of three and 158 involving children aged three or older. The interviews were conducted at three AMAs (Jaguaré, Paulo VI, and Vila Sônia) and three PHCUs/FHS (Jardim D'Abril, Vila Dalva, and Jardim Boa Vista); at each of the specified locations, caregivers of 52 children were interviewed, with the exception of PHCU/FHS Jardim Boa Vista, where 51 assessments were conducted. Table 1 presents information about the sociodemographic data of the children and their caregivers, organized according to age groups.

**Table 1:** General characteristics of the studied sample according to the child's age range.

	< 3-years (*n* = 153)	≥ 3-years (*n* = 158)	Total (*n* = 311)	p
	Mean ± SD/ n (%)	Mean ± SD/ n (%)	Mean ± SD/ n (%)	
**Child's Information**				
Female	84 (54.9%)	87 (55.1%)	171 (55.0%)	1
**Caregiver's Information**				
Age	28.8 ± 7.9	33.1 ± 9.7	31.0 ± 9.1	0.000^a^
Female	142 (92.8%)	149 (94.3%)	291 (93.6%)	0.760
**Economy Class**				0.769
A or B	27 (17.6%)	33 (20.9%)	60 (19.3%)	
C	112 (73.2%)	111 (70.3%)	223 (71.7%)	
D or E	14 (9.2%)	14 (8.9%)	28 (9%)	
**Education level**				0.004^a^
Incomplete elementary school	6 (3.9%)	4 (2.5%)	10 (3.2%)	
Complete elementary school	16 (10.5%)	43 (27.2%)	59 (19.0%)	
Complete middle school	49 (32.0%)	43 (27.2%)	92 (29.6%)	
Complete high school	77 (50.3%)	61 (38.6%)	138 (44.4%)	
Bachelor's degree	5 (3.3%)	7 (4.4%)	12 (3.9%)	

a *p* < 0.05.

The results from Table 1 indicate a balanced representation of both sexes among the children in this study. Caregivers, for the most part, are female and with a mean age of 31 years. The majority of caregivers have a satisfactory level of education. However, it is important to note that 22% of them have only completed Elementary School. Caregivers of younger children have higher levels of education compared to caregivers of children older than three years of age. Additionally, they are also younger than their counterparts as well.

Table 2 shows the comparison of PCA Tool scores between age groups. It is observed that the scores evaluating affiliation, longitudinality, coordination, and completeness have higher scores in the group of children under three years of age, in both types of healthcare services, with statistically significant differences. These values influence the scores of the essential and overall scores. However, it is important to note that these scores do not reach the ideal cutoff point, which is greater or equal to 6.6.

**Table 2:** Evaluation of PCA Tool scores on a scale of 0 to 10 points between the groups < and ≥ 3-years.

	< 3-years (*n* = 153)	≥ 3-years (*n* = 158)	Total (*n* = 311)	p
	mean±SD/ n (%)	mean±SD/ n (%)	mean±SD/ n (%)	
A Score (Affiliation)	4.9 ± 4.3	3.8 ± 3.9	4.3 ± 4.1	0.021^a^
B Score (Access - utilization)	7.2 ± 2.8	7.4 ± 2.5	7.3 ± 2.7	0.655
C Score (Access - accessibility)	5.8 ± 2.5	5.3 ± 2.4	5.6 ± 2.4	0.058
D Score (Longitudinality)	5.7 ± 1.7	5.2 ± 1.8	5.4 ± 1.8	0.006^a^
E Score (Coordination - Care Integration)	7.5 ± 3.4	7.4 ± 3.3	7.4 ± 3.3	0.886
F Score (Coordination - Information Systems)	7.4 ± 2.0	6.3 ± 2.7	6.8 ± 2.4	0.000^a^
G Score (Completeness - Available Services)	6.1 ± 2.5	5.7 ± 2.8	5.9 ± 2.6	0.195
H Score (Completeness - Services Provided)	5.4 ± 3.7	4.5 ± 3.9	4.9 ± 3.8	0.043^a^
I Score (Family Orientation)	4.8 ± 3.4	5.0 ± 3.3	4.9 ± 3.3	0.679
J Score (Community Orientation)	7.2 ± 4.1	6.9 ± 4.2	7.0 ± 4.2	0.606
Essential Score	6.1 ± 2.0	5.5 ± 2.0	5.8 ± 2.0	0.008^a^
Overall Score	6.0 ± 2.2	5.6 ± 2.2	5.8 ± 2.2	0.054

a *p* < 0.05.

Table 3, Table 4 present the assessment of scores between the age groups studied, considering the type of healthcare service, AMA or PHCU/FHS. When analyzing these tables, it is noticeable that, for both services, the score “Coordination - Information Systems” exhibits a statistically significant difference between the different age groups. This suggests that younger children have a greater number of health-related records, possibly due to more frequent visits to healthcare services.

**Table 3:** The assessment of PCA Tool scores on a scale of 0 to 10 points between the groups < 3-years and ≥ 3-years by interview location – PHCU/FHS.

	< 3-years PHCU/FHS (*n* = 87)	≥ 3-years PHCU/FHS (*n* = 68)	Total (*n* = 155)	p
A Score (Affiliation)	7.9 ± 2.8	6.8 ± 3.5	7.4 ± 3.2	0.040^a^
B Score (Access- utilization)	7.9 ± 2.5	7.9 ± 2.2	7.9 ± 2.3	0.984
C Score (Access- accessibility)	6.3 ± 2.6	5.7 ± 2.7	6.1 ± 2.6	0.141
D Score (Longitudinality)	6.6 ± 1.3	6.3 ± 1.6	6.5 ± 1.5	0.234
E Score (Coordination - Care Integration)	8.6 ± 2.4	9.0 ± 2.0	8.8 ± 2.2	0.587
F Score (Coordination - Information Systems)	7.9 ± 1.5	7.3 ± 2.0	7.6 ± 1.8	0.029^a^
G Score (Completeness- Available Services)	7.1 ± 1.6	7.1 ± 1.9	7.1 ± 1.7	0.960
H Score (Completeness - Services Provided)	7.1 ± 3.1	6.8 ± 3.4	6.9 ± 3.2	0.604
I Score (Family Orientation)	6.0 ± 3.3	6.9 ± 2.9	6.4 ± 3.1	0.101
J Score (Community Orientation)	9.5 ± 3.2	9.9 ± 3.1	9.7 ± 3.1	0.438
Essential Score	7.3 ± 1.4	6.9 ± 1.8	7.1 ± 1.6	0.106
Overall Score	7.3 ± 1.5	7.2 ± 1.8	7.3 ± 1.7	0.607

a *p* < 0.05.

**Table 4:** The assessment of PCA Tool scores on a scale of 0 to 10 points between the groups < 3-years and ≥ 3-years by interview location – AMA.

	< 3-years AMA (*n* = 66)	≥ 3-years AMA (*n* = 90)	Total (*n* = 156)	p
A Score (Affiliation)	0.9 ± 2.0	1.5 ± 2.2	1.2 ± 2.1	0.086
B Score (Access- utilization)	6.4 ± 3.1	7.0 ± 2.7	6.8 ± 2.9	0.215
C Score (Access- accessibility)	5.2 ± 2.1	5.0 ± 2.1	5.1 ± 2.1	0.621
D Score (Longitudinality)	4.5 ± 1.3	4.3 ± 1.3	4.4 ± 1.3	0.288
E Score (Coordination - Care Integration)	5.4 ± 4.2	5.4 ± 3.5	5.4 ± 3.6	0.998
F Score (Coordination - Information Systems)	6.6 ± 2.3	5.5 ± 2.9	6.0 ± 2.7	0.014^a^
G Score (Completeness- Available Services)	4.6 ± 2.8	4.5 ± 2.8	4.6 ± 2.8	0.860
H Score (Completeness - Services Provided)	3.1 ± 3.3	2.8 ± 3.4	2.9 ± 3.3	0.505
I Score (Family Orientation)	3.2 ± 2.9	3.6 ± 2.8	3.4 ± 2.8	0.461
J Score (Community Orientation)	4.3 ± 3.3	4.4 ± 3.3	4.4 ± 3.3	0.853
Essential Score	4.5 ± 1.5	4.4 ± 1.5	4.4 ± 1.5	0.791
Overall Score	4.3 ± 1.6	4.3 ± 1.5	4.3 ± 1.5	0.990

a *p* < 0.05.

In Table 3, the affiliation attribute also showed a significantly higher score in the group of children under three years old.

Table 5 presents the construction of a linear regression model to assess the relationship between essential and overall scores and the type of healthcare unit, considering the age group of the children. The model was adjusted for the age of the caregiver (equal to or greater than 30 years), the sex of the child and the caregiver, and the caregiver's education level (using completion of high school as the cutoff point). It is noteworthy that the essential score for PHCU/FHS shows a tendency to have lower values in the assessments of children over three years of age.

**Table 5:** Beta coefficient for essential and overall scores of the PCA Tool, associated with children aged ≥ 3-years.

		PHCU/FHS	AMA
**Essential Score**	Crude	−0.420 (−0.911 to 0.071) *p* = 0.095	−0.064 (−0.537 to 0.409) *p* = 0.791
	Adjusted	−0.400 (−0.919 to 0.120) *p* = 0.134	−0.024 (−0.507 to 0.458) *p* = 0.921
**Overall Score**	Crude	−0.143 (−0.676 to 0.390) *p* = 0.599	0.003 (−0.487 to 0.494) *p* = 0.990
	Adjusted	−0.151 (−0.715 to 0.413) *p* = 0.600	0.061 (−0.438 to 0.561) *p* = 0.810

The model was adjusted for the caregiver's age (equal to or greater than 30-years), child and caregiver's gender, and the caregiver's educational level (using the completion of high school as the cutoff point).

## Discussion

This study is the first to comparatively assess the attributes of PHC using the PCA Tool instrument across pediatric age groups, considering the age of three as the cutoff point (children under three years old and those three years and older). This cutoff point ensured a balanced distribution of age groups in the samples of children in AMAs and PHCUs, as well as allowed an evaluation of early and late childhood. During this period, children exhibit significant gains in both growth and development and access health services very frequently. [27]

Comparative results between age groups show higher essential and overall scores in the assessment of children under three years old, with the essential score significantly better for this phase of life (6.1 compared to 5.5, as shown in Table 2). This indicates that services offered for early childhood are positively recognized by the population, although this score does not reach the ideal value of 6.6, according to the literature. [13,20,28,29] The authors also observed that the scores that showed statistically significant differences in both groups (under three years old and three years and older) were the following: affiliation (4.9 vs. 3.8), continuity of care (5.7 vs. 5.2), coordination - information system (7.4 vs. 6.3), and completeness - services provided (5.4 vs. 4.5).

Although it did not reach the ideal score, continuity of care was higher in younger children, indicating that the follow-up proposed by the Ministry of Health, with scheduled appointments, is being implemented and recognized as important by the population. From the age of three, the lower score for this attribute reflects that child health follow-up becomes primarily on-demand, meaning that care focuses on acute illnesses and is less effective in promoting health. It is remarkable that the increase in non-communicable chronic diseases occurs insidiously and is noticed late by caregivers, making it difficult to provide guidance on lifestyle habits and the management of comorbidities. Other studies in the literature, regardless of age group analysis, have also found that healthcare units perform poorly in terms of preventing illnesses and promoting health. [30]

Similarly, to the previous discussion, the same analysis can be applied to the attribute completeness - services provided, which assesses the services offered or received. In this context, issues such as healthy eating, hygiene, sleep, accident prevention, as well as child behavior and development, are more comprehensively addressed in scheduled and frequent appointments.

The attribute coordination - information system obtained a score above the ideal for children under three years of age in this study. This may indicate that in the evaluated units, where Family Medicine residency programs are in place, clinical records are an integral part of the training of these professionals. Ensuring adequate information is crucial for the safety of childcare, and several studies [31,32] have shown that recording patient information plays a crucial role both in the appointment itself and in understanding the individual's health progress.

In the group of children under 3 years old, in Table 2, the affiliation attribute obtained a higher score. However, a more accurate understanding can be gained from Table 3, as the high scores observed in Table 2 result from the evaluation of this attribute in the PHCU/FHS, not in the AMA. The degree of affiliation reflects the perception of this health service/professional as fundamental in promoting user care.

Of the four most recent studies 33, 34, 35, 36 that assessed affiliation, three of them presented scores above 6.6. 33, 34, 35 One of these studies was conducted with caregivers of children with Type 1 Diabetes Mellitus, [35] a chronic condition that presupposes a strong bond between the user and the responsible professional. The most methodologically well-designed study, more accurately representing the assessment of children's health, was conducted in the city of Rio de Janeiro, where the children's experience with FHS services was well evaluated, achieving a score of 7.59. [33]

In Table 3, Table 4, when considering both age group and type of service (PHCU/FHS), it is noted that the attribute coordination - information system obtained a higher score in the group of children under three years old. Based on these data, the authors may speculate that the medical record is recognized by the child's guardian as an effective tool for both acute demands and longitudinal follow-up, especially during early childhood. This is supported by Silva et al.’s results. [37] In their study, when assessing the perception of caregivers of children up to two years of age who exclusively attended PHCUs/FHS, they found a high score for this attribute.

In the analysis of attributes between age groups in relation to the location of care (PHCU/FHS), several high scores can be observed. Although there is no statistical difference between the groups, the highest values are related to coordination - care integration (8.6 vs. 9.0) and community orientation (9.5 vs. 9.9). The result of the community orientation attribute stands out, as it does not coincide with what was found in other studies. [37,38] It is inferred from this result that the work of the Family Health Strategy in the development of collective health has been extremely satisfactory for both age groups and can play an important role in preventing non-communicable chronic diseases for preschool and school-age children.

Given the proposal to create AMAs [39] with the aim of increasing access to PHC, this study revealed that the attribute access - utilization performed satisfactorily in both age groups, exceeding the ideal value when assessed in the group of children aged three and older (7.0, as shown in Table 4). However, when analyzing the accessibility aspect of access, which indicates service structure, location, and hours, this score was insufficient in both groups, confirming findings from a previous study. [39]

The other attributes received insufficient evaluations in both age groups, with a maximum of 6.6 for the coordination-information system parameter in the group of children under three years old. These results demonstrate concerns regarding the effectiveness and quality of the service provided.

Regarding the sociodemographic profile of the studied population, it is observed that children under three years of age were predominantly cared for by young women with higher education compared to the other group (Table 1). This can lead to different perspectives and perceptions regarding the studied attributes.

In linear regression models, the authors observed a non-significant trend towards better overall and essential scores in the evaluations of the group of children under three years old attending PHCUs/FHS. Although no statistical significance was observed, this result may reflect that strategies for early childhood are positively recognized by the population, according to the PCATool domains, especially when it comes to PHCUs/FHS.

Current literature points to other opportunities for implementing nutritional and developmental programs for preschoolers, school-age children, and adolescents. [40] From this perspective, healthcare units have failed to recognize and manage health issues related to nutritional problems, such as overweight, as well as deficits and childhood developmental disorders. This situation is exacerbated by the lack of population awareness regarding these conditions, making it imperative to adopt a new approach in terms of public policies for children beyond early childhood.

One limitation of this research is that the results represent a privileged area of health access and, above all, rely on highly qualified professionals. This is because all the healthcare units studied have residency programs in Family Medicine at the University of São Paulo. This bias may have positively influenced the essential and overall scores related to PHCUs/FHS (Table 3), which does not correlate with the national reality. AMA is a service exclusive to the city of São Paulo and is less studied compared to more traditional PHC services. Although the AMA is classified as part of Primary Health Care and, therefore, was evaluated by the PCA Tool, specific aspects of this strategy may require evaluation by other instruments.

Other limitations are related to the lower scores observed in various attributes evaluated in the AMA service, impacting the averages presented in Table 2. Additionally, the data were collected within UBSs and AMAs, which could introduce bias in the scores of items related to access (B and C).

## Conclusion

The attributes of PHC, with a focus on affiliation, continuity of care, coordination, and completeness, received more favorable assessments in the group of children under three years of age when considering all services, including AMAs and PHCUs/FHS. When analyzing the services separately, it was observed that the coordination-information system attribute scored higher in the group of children under three years old. Additionally, in the PHCU/FHS service, the affiliation attribute also recorded higher scores in the same group of children.

The linear regression analysis indicated a trend of lower essential scores when evaluating children over three years of age.

This study suggests the system of care for children aged three years or older may need priority improvement in response to new health demands.

## CRediT authorship contribution statement

**Luciana Harumi Miranda Omori:** Conceptualization, Resources, Visualization, Writing – original draft. **Deoclecio Avigo:** Software, Investigation, Resources, Visualization. **Itamar de Souza Santos:** Conceptualization, Methodology, Software, Formal analysis. **Gustavo Diniz Ferreira Gusso:** Conceptualization, Methodology, Funding acquisition. **Maria Teresa Bechere Fernandes:** Conceptualization, Formal analysis, Writing – review & editing.

## Conflicts of interest

The authors declare no conflicts of interest.

## Appendix Supplementary materials

Image, application 1
