# Supplementary material for: Comparative analysis of primary health care attributes between children under and over 3 years of age using the primary care assessment tool
Source: Clinics (Sao Paulo). 2024 Apr 4;79:100353. doi: 10.1016/j.clinsp.2024.100353 (PMC11001613; doi:10.1016/j.clinsp.2024.100353)
Supplement: Supplementary file 1 [file mmc1.docx]

Abstract
Child health actions in Brazil have their primary focus on early childhood. A new epidemiological profile is emerging for children after the first one thousand days: an increase in non-communicable chronic diseases. This research aimed to analyze the attributes of Primary Health Care comparatively among different age groups, using three years of age as the cutoff point. Methods The study design was cross-sectional and conducted in three Primary Health Care Units and three Ambulatory Medical Assistance facilities in the Western Region of São Paulo. The PCA Tool Brazil was used as the assessment instrument. Results A total of 311 interviews were conducted with caregivers of children aged 0 to 12 years; 153 children were under three years old, and 158 were three years or older. The attributes that showed statistically significant differences between age groups (< 3 years and > 3 years) were: affiliation (4.9 x 3.8), longitudinality (5.7 x 5.2), information system (7.4 x 6.3), and services provided (5.4 x 4.5). Through linear regression analysis, it was observed that there was a trend for better overall and essential scores in the evaluations of the group of children under three years old who attended Primary Health Care Units. Conclusion The comparative analysis of Primary Health Care attributes among pediatric age groups revealed a trend towards higher scores, according to caregivers' perceptions, for children under three years old. This study suggests the need for the implementation of programs that can better address the healthcare needs of children beyond early childhood

Keywords: Primary Health Care, PCA Tool, Child Health
